# Novel Magnetic Cross-Linked Cellulase Aggregates with a Potential Application in Lignocellulosic Biomass Bioconversion

**DOI:** 10.3390/molecules22020269

**Published:** 2017-02-10

**Authors:** Junqi Jia, Weiwei Zhang, Zengjie Yang, Xianling Yang, Na Wang, Xiaoqi Yu

**Affiliations:** 1 Key Laboratory of Green Chemistry Technology, Ministry of Education, College of Chemistry, Sichuan University, Chengdu 610064, China; scujunqi@163.com (J.J.); 15864374098@163.com (Z.Y.); 13550337266@163.com (X.Y.); 2 School of Chemistry and Chemical Engineering, Ningxia University, Yinchuan 750021, China; zhangww@nxu.edu.cn

**Keywords:** cellulase, magnetic nanoparticles, cross-linked enzyme aggregates, hydrolysis, biomass

## Abstract

The utilization of renewable biomass resources to produce high-value chemicals by enzymatic processes is beneficial for alternative energy production, due to the accelerating depletion of fossil fuels. As immobilization techniques can improve enzyme stability and reusability, a novel magnetic cross-linked cellulase aggregate has been developed and applied for biomass bioconversion. The cross-linked aggregates could purify and immobilize enzymes in a single operation, and could then be combined with magnetic nanoparticles (MNPs), which provides easy separation of the materials. The immobilized cellulase showed a better activity at a wider temperature range and pH values than that of the free cellulase. After six cycles of consecutive reuse, the immobilized cellulase performed successful magnetic separation and retained 74% of its initial activity when carboxylmethyl cellulose (CMC) was used as the model substrate. Furthermore, the structure and morphology of the immobilized cellulase were studied by Fourier transform infrared spectroscopy (FTIR) and scanning electron microscopy (SEM). Moreover, the immobilized cellulase was shown to hydrolyze bamboo biomass with a yield of 21%, and was re-used in biomass conversion up to four cycles with 38% activity retention, which indicated that the immobilized enzyme has good potential for biomass applications.

## 1. Introduction

Lignocellulosic biomass, the most abundant and bio-renewable resource on the Earth, has attracted worldwide attention with the limitation of fossil fuels and environmental concerns [1]. One of the most important steps for the application of biomass is the hydrolysis of cellulose to glucose, after which the glucose can be used for the production of ethanol or other chemicals as energy sources [2,3]. However, it remains a challenge due to the complicated and robust structure of lignocellulosic biomass. Most lignocellulosic biomass mainly contains 35%–50% of cellulose, 20%–35% of hemicellulose, and 10%–25% of lignin [1]. Hemicelluloses are branched polymers, which consist of a range of different sugars through glycosidic bonds and are embedded in cellulose elementary fibrils. Lignin is a complicated heterogeneous polymer of phenylpropane units linked by ether and carbon-carbon bonds [4]. Obviously, these polymers in the biomass have been cited as barriers and impact catalysts’ access to cellulose. Moreover, cellulose is a linear and syndiotactic polymer of β-d-glucose, and its β-1,4-glycosidic bonds are tightly linked via intra- and intermolecular hydrogen bonds, hindering the accessibility of the catalysts to the chains and simultaneously making cellulose insoluble in conventional solvents. Hence, it is important to develop an effective method to make the catalysts accessible to cellulose and break the chemical bonds to obtain glucose [5].

Many approaches have been studied to obtain water-soluble sugars by using homogeneous and heterogeneous catalysts such as liquid acids [6,7], solid acids [8,9,10,11], and enzymes [12,13]. Though the acid catalyzed hydrolysis is efficient for glucose production, there are still several problems to be solved, such as unavoidable side-reactions, harsh conditions, product purification, equipment corrosion, waste effluent treatment, and so on. Therefore, it is necessary to seek a greener and more efficient method for converting cellulose to glucose [1]. Among these methods, hydrolysis catalyzed by cellulase has shown an attractive application potential, since it has mild reaction conditions, high yield conversion, and is environmentally friendly [14]. Cellulase is a common collective term for a mixture of three different enzymes, namely endoglucanases (EC 3.2.1.4), cellobiohydrolases (EC 3.2.1.91), and β-glucosidases (EC 3.2.1.21). These enzymes work synergistically to produce glucose which is subsequently fermented into ethanol. Nevertheless, there are several challenges facing commercially viable enzyme catalyzed applications, such as low stability, high enzyme costs, and barriers to scale up [15]. Enzyme immobilization, the localization of enzymes within a defined matrix or support, is a well-established technology, which promisingly improves enzyme properties such as stability, activity, and selectivity. Generally, the immobilization of enzymes on a support may permit the enzyme molecules to be fully dispersed with no possible aggregation, and the covalent attachment between the support and the enzyme promotes a rigidification of the enzyme structure, which preserves enzyme properties under drastic conditions and reduces enzyme inhibitions, thus increasing the enzyme stability and preserving enzyme activity. If we control the medium, the effector, or the support in a “rational” way, the enzyme will congeal a structure with better activity or selectivity via immobilization, therefore improving the economic viability of the process [16,17]. Previous studies demonstrated that the immobilization of cellulase can improve the stability and reusability in various degrees [18,19,20,21,22,23,24,25,26]. For example, the immobilization of cellulase on magnetic nanoparticles (MNPs) by the adsorption method can retain the structure of enzyme very well, but the weak interaction may lead to the leakage of the enzyme from the support, which influences the stability and reusability [21]. Compared to adsorption, although the strong interaction involving covalent binding may result in some loss of activity, it generally provides a more stable attachment, thus preventing the leakage of the enzyme [22,23,24].

The properties of the immobilized enzyme are significantly influenced by the selection of the immobilization method. Among different immobilization methods, cross-linked enzyme aggregates (CLEAs) have emerged as novel and versatile biocatalysts, which are easily prepared and are effective with high stability [27,28]. However, CLEAs may be considered too soft for industrial applications in reactor configuration and are difficult to be separated from the reaction mixture for re-use, hence hindering their applications [29]. The further immobilization of CLEAs on supports with better mechanical properties has been proposed as a good solution, such as trapping CLEAs in LentiKats [30], or trapping them in a membrane [31]. In recent years, the utilization of nanomaterials as supports for enzyme immobilization has generated great interest in the biochemistry and biomedical fields due to their extraordinary properties compared to the bulk materials. These robust nanostructured forms have larger surface area-to-volume ratios and subsequent higher enzyme loading, lower mass transfer resistance, and good biocatalytic potential. A variety of nanomaterials have been employed for enzyme immobilization, such as nanoparticles, nanofibres, nanotubes, nanoporous, nanosheets, and nanocomposites [15,32]. Among these, the study of MNPs has grown rapidly due to their strong magnetic properties, ease of separation, lower mechanical shearing and process costs, and improvement of the operational stability of the enzyme, thus making them more competitive for enzyme immobilization [33,34]. Therefore, a novel magnetic cross-linked aggregates method for immobilization has been investigated. In recent years, Talekar et al. developed magnetic CLEAs of α-amylase to enhance the stability and reusability of enzymes [35]. Recently, we also prepared surfactant activated magnetic lipase CLEAs and applied them in continuous biodiesel production with good potential [36].

Herein, for the first time we combine the precipitated cross-linked cellulase aggregates with magnetic nanoparticles. In the present work, magnetic iron oxide nanoparticles (Fe_3_O_4_) were utilized and modified by 3-aminopropyltriethoxysilane to provide functional groups and prevent the excessive aggregation of Fe_3_O_4_. Then, cellulase was immobilized on the modified particles with glutaraldehyde. The size and structure of the immobilized enzyme were investigated, and its stability at wider pH and temperature ranges were measured. Moreover, to the best of our knowledge, only a few studies of cellulase immobilization have focused on the hydrolysis of lignocellulosic biomass. Recently, the *Phyllostachys heterocycla* cv. var. *pubescens* (the common name bamboo is used hereafter) has shown potential for industrial exploitation because of its worldwide distribution and fast growth [37,38]. In this work, as a typical lignocellulosic biomass, this bamboo was pretreated and hydrolyzed by immobilized cellulase.

## 2. Results and Discussion

### 2.1. Chemical Characterisation and Analysis

**Figure 1:**
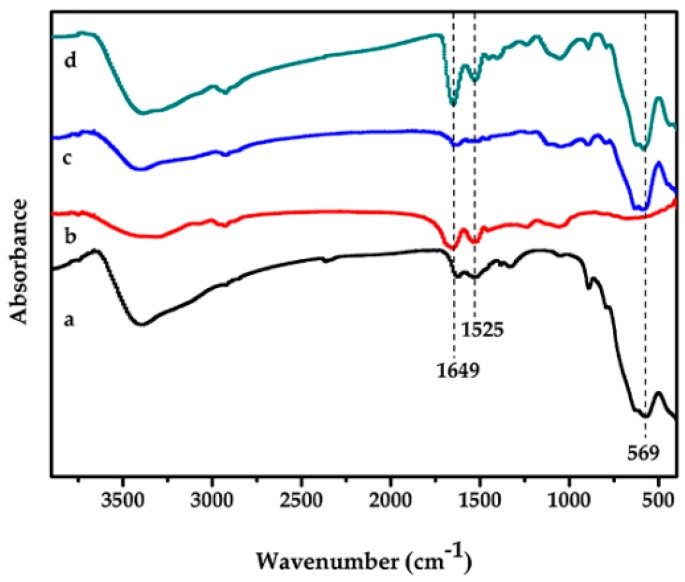
Fourier transform infrared (FTIR) spectra of (**a**) Fe_3_O_4_; (**b**) cellulase-cross-linked enzyme aggregates (CLEAs); (**c**) 3-aminopropyl triethoxysilane (APTES)-Fe_3_O_4_; (**d**) magnetic-cellulase-CLEAs.

The Fourier transform infrared spectroscopy (FTIR) spectra of Fe_3_O_4_, modified Fe_3_O_4_, cellulase-CLEAs, and magnetic-cellulase-CLEAs are given in Figure 1. A stretch in the peak at 569 cm^−1^ corresponds to the Fe–O vibrations of the magnetite core [39], and the characteristic peak also appeared in Figure 1c,d, which indicated the structure of Fe_3_O_4_ was preserved after chemical modification. The characteristic peaks at 1649 cm^−1^ and 1525 cm^−1^ represent the amide I (C=O stretching vibrations) and amide II (N–H bending and C–N stretching) of cellulase, respectively (Figure 1b). The spectra of the magnetic-cellulase-CLEAs showed similar peaks of amide bands, which indicated that cellulase was successfully attached to the support. The obtained peaks all well matched those in earlier reports [23,40].

**Figure 2:**
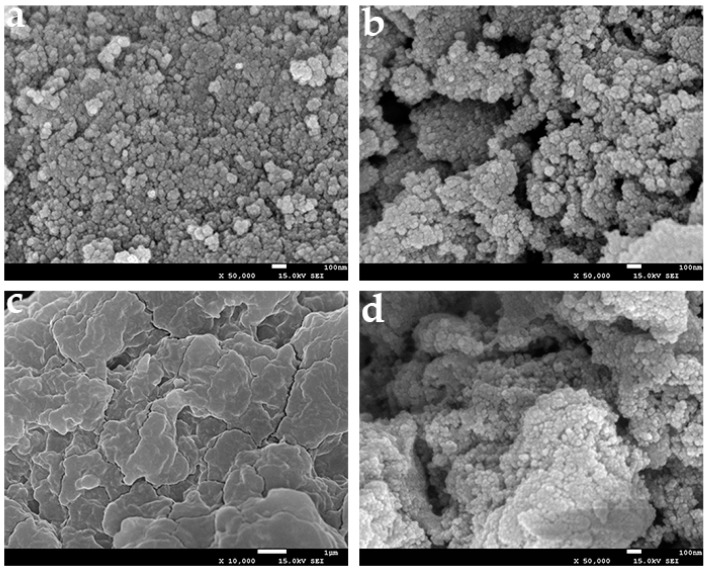
Scanning electron microscopy (SEM) images of (**a**) Fe_3_O_4_; (**b**) APTES-Fe_3_O_4_; (**c**) cellulase-CLEAs; (**d**) magnetic-cellulase-CLEAs.

The surface morphology and size of the Fe_3_O_4_ particles, 3-aminopropyl triethoxysilane (APTES)-Fe_3_O_4_, and immobilized cellulase were also observed by scanning electron microscopy (SEM) (Figure 2). The average diameters of the Fe_3_O_4_ particles ranged from 20 to 60 nm, and such nanometer size is beneficial for loading more enzyme since they have a larger specific surface area. As shown in Figure 2a, the Fe_3_O_4_ particles were arranged tightly, and a looser surface structure of APTES-Fe_3_O_4_ particles was observed after modification (Figure 2b). As shown in Figure 2c, the cellulase-CLEAs were larger than the Fe_3_O_4_ particles. As for the magnetic-cellulase-CLEAs, the modified Fe_3_O_4_ particles were added before enzyme precipitation, thus the MNPs could act as cores during the cellulase precipitation, and also cross-link with the enzyme. Therefore, as shown in Figure 2d, the magnetic-cellulase-CLEAs had a larger specific surface area than that of cellulase-CLEAs, and the volumetric active sites were also increased.

### 2.2. Optimal Conditions for the Immobilization of Cellulase

**Figure 3:**
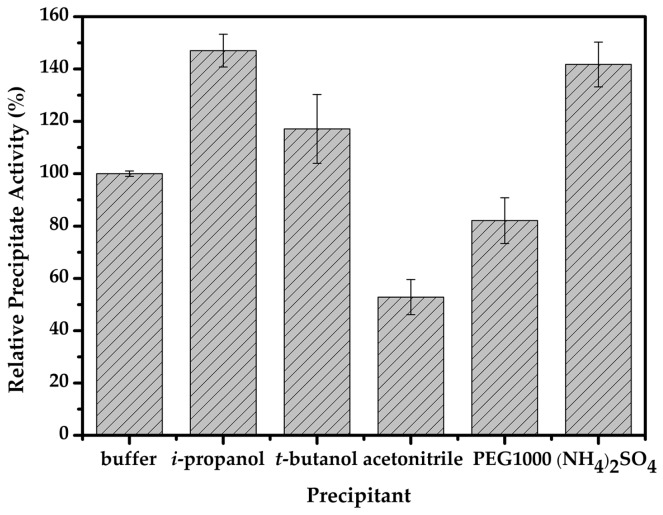
Precipitation of cellulase with different precipitants.

The precipitation step predictably has a crucial effect on the activity in CLEAs as it may precipitate the target enzyme under appropriate conditions, and causes physical aggregation of enzyme molecules into supramolecular structures, which are subsequently cross linked to lock enzymes in CLEAs. Generally, salts, water-miscible organic solvents, and non-ionic polymers have been used as precipitants. Here, five typical protein precipitating agents such as *iso*-propanol, *tert*-butanol, acetonitrile, polyethylene glycol 1000 (PEG1000), and saturated ammonium sulfate were investigated. As shown in Figure 3, the activity of the magnetic-cellulase-CLEAs without precipitant was defined as 100%, and different types of precipitants affected the activity of the immobilized cellulase to various degrees. The *iso*-propanol exhibited the best enzyme activity and improved almost 50% with respect to the CLEAs without precipitant, following saturated ammonium sulfate and *tert*-butanol. PEG1000 showed a lower enzyme activity recovery, which may because of the denaturation under aggregation and the cross-linking step, and it worsened when acetonitrile was used. The results indicated the significant role of the precipitant in the immobilization step; in consideration of the appropriate precipitant for the immobilized cellulase, *iso*-propanol was used in subsequent experiments.

**Figure 4:**
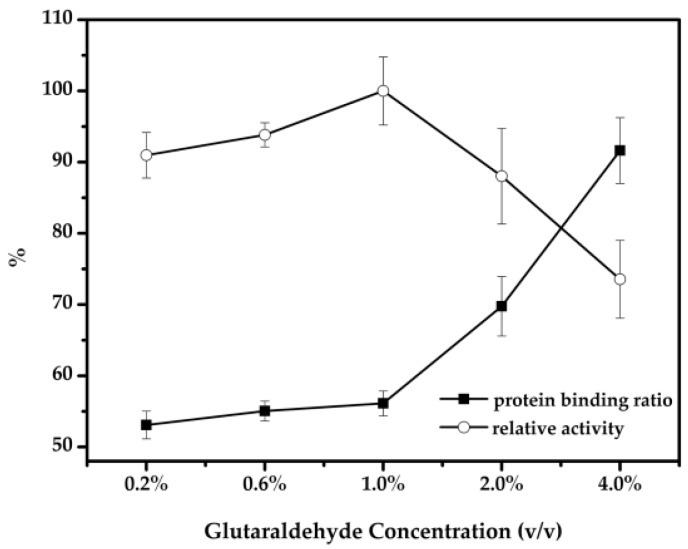
Effect of the glutaraldehyde concentration on the activity of immobilized cellulose.

Traditionally, glutaraldehyde is a commonly used cross-linking agent in enzyme immobilization, and the concentration of the cross linker is one key parameter of CLEAs as it influences the activity, stability, and particle size of the resulting CLEAs. Here, different concentrations of glutaraldehyde were investigated to obtain effective cross-linking and recycling. As shown in Figure 4, with the increase in concentration of glutaraldehyde, the activity of the immobilized cellulase presented a bell-shaped curve, and the best relative activity of magnetic-cellulase-CLEAs was achieved when the concentration of glutaraldehyde was 1.0% *v/v*. At lower cross linker concentrations, insufficient cross linking occurred, resulting in operationally unstable CLEAs releasing enzyme into the reaction medium. However, excessive glutaraldehyde could decrease the enzyme activity [41], because glutaraldehyde is also a protein denaturant which may cause significant change in the protein structure. Additionally, the flexibility of the enzyme may be limited, and the rigidification of the enzyme prevents the substrate from reaching the active site, thus increasing the steric hindrance [27]. Therefore, to achieve an efficient and active immobilized enzyme, an optimum cross linker concentration should be used.

**Figure 5:**
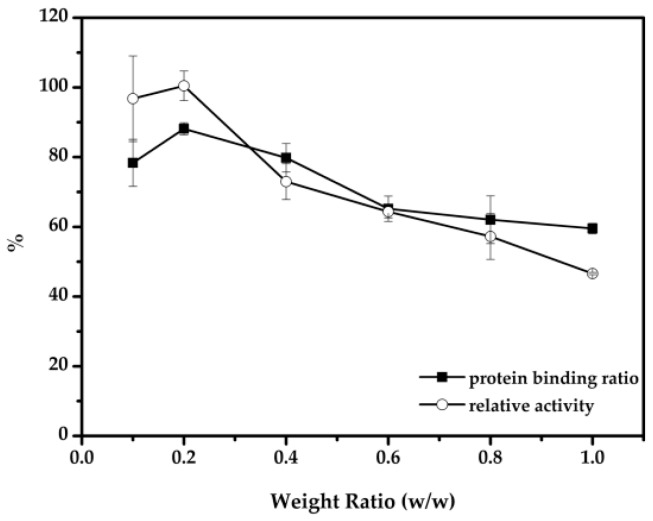
Effect of the weight ratio (cellulase to nanoparticles) on the activity of immobilized cellulose.

To achieve effective immobilization, the effect of the cellulase-to-nanoparticle ratio was also studied. As can be seen from Figure 5, the activity of the immobilized enzyme, as well as the protein binding ratio, decreased as the amount of cellulase used increased, which was similar to the previous studies [42]. Excessive protein may form compact aggregates [43], which could lead to a loss of flexibility and could hinder active sites from attaching to substrates. The optimal weight ratio of cellulase and nanoparticles was 0.2 with a protein binding ratio reaching 88%, and the cellulase immobilized on the supports was 176 mg/g.

### 2.3. Effect of pH and Temperature for the Immobilized Cellulase

**Figure 6:**
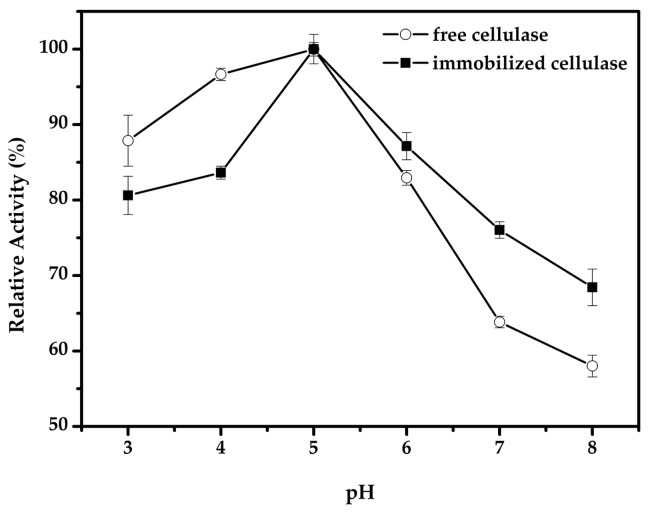
Effect of pH on the activity of immobilized cellulase.

The effect of pH on the activity of free and immobilized cellulase was performed in the pH range of 3–8 at 50 °C. As shown in Figure 6, the free and immobilized cellulase both achieved maximum activity at pH 5.0. When the pH condition was above 5.0, the immobilized cellulase showed a significantly higher activity retention compared to the free cellulase. As the ionic groups within the cellulase molecule produced a strong electrostatic repulsion under relatively alkaline media, the immobilization of cellulase in magnetic CLEAs could prevent the destruction and degeneration of the enzyme active center due to the effect of tight covalent bonding. A similar observation has been reported with cellulase immobilized on magnetic chitosan nanoparticles [23]. However, another study found there was a shift in optimum pH values with immobilized β-glucosidase on Eri silk fibrion particles via an adsorption method; this change may due to the charged groups’ interactions between the enzyme and carrier [44].

**Figure 7:**
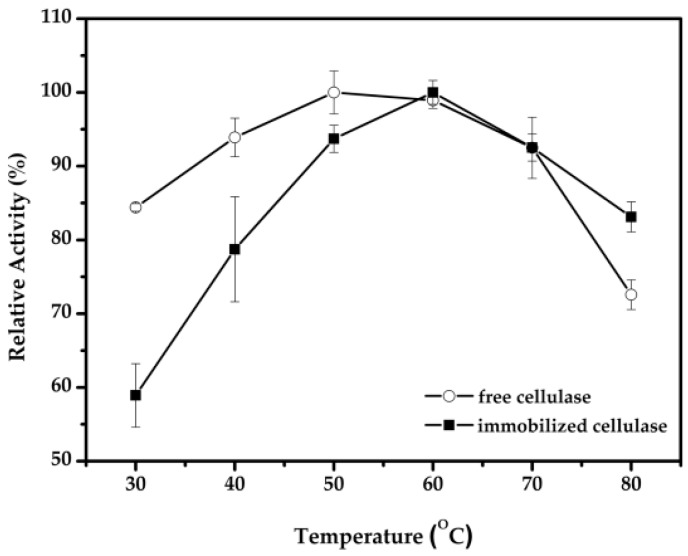
Effect of temperature on the activity of immobilized cellulase.

The influence of temperature on the activity of cellulase was studied from 30 to 80 °C at pH 5.0 (Figure 7). Obviously, the activities of both free and immobilized cellulase were affected by temperature. Additionally, a shift appeared when the free cellulase reached the maximum activity at 50 °C, whereas the immobilized cellulase reached the maximum activity at 60 °C. It demonstrated that the immobilized cellulase showed good temperature stability compared to that of the free cellulase at higher temperature. This result was consistent with previous studies, where the temperature for the highest alpha amylase activity was established at 45 °C for the free enzyme and shifted to 60 °C for magnetic CLEAs [35]. That may be attributed to the immobilization scaffold, which prevented stretching of the enzyme molecule at higher temperatures. However, the immobilized cellulase performed with lower activity below 60 °C, which indicated that the diffusion of the viscous substrate in magnetic-cellulase-CLEAs was hindered at lower temperatures.

### 2.4. Carboxylmethyl Cellulose Reusability

**Figure 8:**
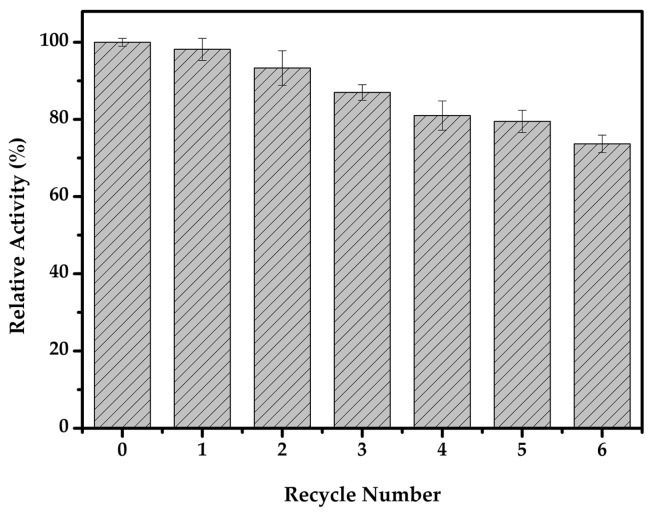
The carboxylmethyl cellulose (CMC) reusability of the immobilized cellulase.

One of the advantages of magnetic nanoparticles is their convenience in handling, ease of separation of the enzyme, and reuse, hence enabling its cost-effectiveness [45]. After three and six consecutive reuses, the immobilized cellulase retained 87% and 74% of its initial activity, showing good reusability (Figure 8). Earlier studies of cellulase immobilization on magnetoreponsive graphene nano-supports retained about 55% of the original activity after four cycles [46]. In another study, the immobilized cellulase, which was covalently bound to the MNPs, maintained about 40% relative activity after six cycles [42]. This gradual decrease in enzyme activity could be due to several factors, such as the leakage of the enzyme from the support, protein denaturation, and product inhibition [24,47]. With its reusability and ease of recovery, the immobilized cellulase would be of great use in industrial applications.

### 2.5. Hydrolysis of Biomass

**Figure 9:**
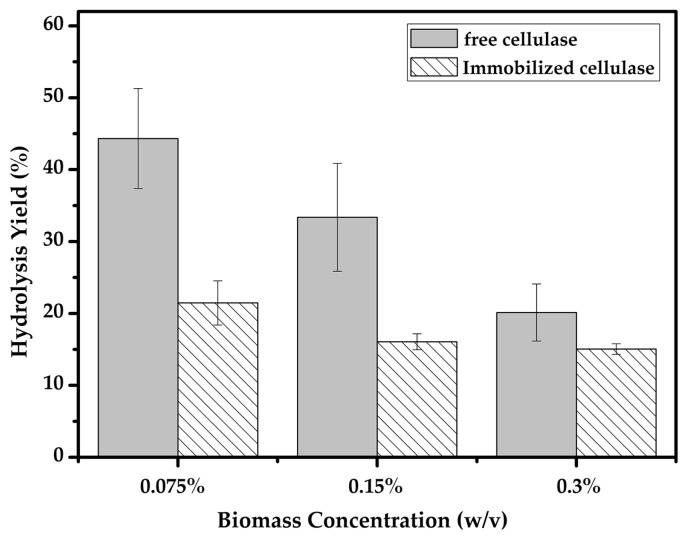
The hydrolysis of pretreated biomass using free and immobilized cellulase.

Varying concentrations of pretreated bamboo were mixed with the same activity of free and immobilized cellulase. As shown in Figure 9, the conversion yield of the hydrolysis reaction was found to reduce with an increasing biomass concentration. The highest hydrolysis yield was achieved at a low biomass concentration, where it reached 44% with the free enzyme and 21% with the immobilized enzyme. However, at higher biomass concentrations, the hydrolysis yield decreased. The change in hydrolysis yield may be due to the substrate accessibility, and to the interference of lignin and hemicellulose with the increase of the biomass. Utilizing the same biomass (*Phyllostachys pubescens*), a study focused on the pretreatment of bamboo chips achieved the maximum enzymatic hydrolysis yield (70.6%) at 72 h [4], and another report achieved 37% for 48 h after optimum pretreatment [48], which demonstrated the bamboo is hard to hydrolyze due to its rigid structure even after pretreatment. The hydrolysis yield of the immobilized enzyme was a little lower than that of the free enzyme, which may due to the limitations of CLEAs, which could form a compact super-molecular immobilized product, therefore, increasing diffusion limitations and leading to a low activity of the immobilized enzyme especially when the substrates were macromolecules [49].

### 2.6. Biomass Hydrolysis Reusability

**Figure 10:**
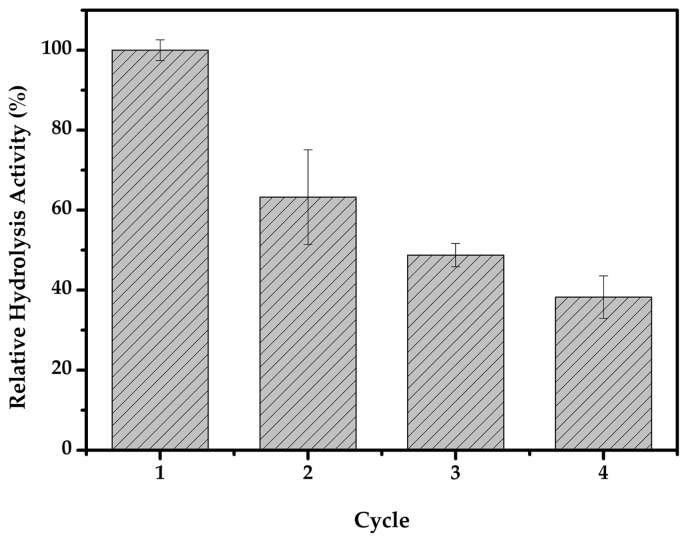
The relative hydrolysis activity of the pretreated biomass using immobilized cellulase.

Reusability is of key importance during the practical application of biocatalysts. Magnetic immobilization offers convenience in handling, ease of separation, and fast reuse of the enzyme, thus enabling its cost-effective use in repeated batches or continuous operations. Figure 10 shows that the hydrolysis activity of immobilized cellulase for two and four cycles was determined to be 63% and 38%, respectively. Although there was a decrease in activity, the results showed the applicability of the magnetic cellulase CLEAs in the hydrolysis of the lignocellulosic biomass. In our earlier research, magnetic cross-linked enzyme aggregates of *Thermomyces lanuginosus* lipase (TLL) were developed, and the immobilized lipase retained 70% of its initial activity after 10 cycles of biotransformation. As for Tween 80-activated TLL-magnetic-CLEAs, they showed no evident decrease of the catalytic activity during the same runs [36]. To achieve more profitable applications, efforts to reach more optimum reaction conditions should be considered, such as pH, temperature, and surfactant choice.

## 3. Materials and Methods

### 3.1. Materials

#### 3.1.1. Enzymes and Chemicals

Cellulase (EC 3.2.1.4; ≥1 U/mg) from *Trichoderma reesei*, 3,5-dinitrosalicylic acid (DNS) were purchased from Sigma (St. Louis, MO, USA). 3-Aminopropyl triethoxysilane and glutaraldehyde (25%, *v/v*) were obtained from Aladdin (Shanghai, China). All other chemicals used were of analytic grade and were available from commercial sources. The water used throughout this work was double distilled water.

#### 3.1.2. Cellulosic Biomass

The *Phyllostachys heterocycla* cv. var. *pubescens* (Anji county of Zhejiang Provice, China) was kindly provided by Prof. Hu (College of Chemistry, Sichuan University, Chengdu, China). The biomass samples were ground to 80 mesh, washed with distilled water three times, and dried at 110 °C in an oven overnight before use. The main components of the dried bamboo were 25.4 wt % lignin, 17.9 wt % hemicellulose, and 46.5 wt % cellulose [37]. As mentioned above, the complex structure of biomass leads to a large challenge for the application of fuels and chemicals. Therefore, an appropriate pretreatment should be employed to remove the lignin and hemicellulose from the substrate, making the structure looser, in order to increase the accessibility of the cellulase to cellulose. The biomass pretreatment was performed by a novel method by He et al. [50]. The bamboo was put in a 200 mL stainless steel autoclave equipped with a magnetic stirring device and a temperature controller. Bamboo powder (3.0 g) and a designated amount of oxalic acid were arranged in the reactor with 100 mL ethanol/H_2_O (*v/v*, 1:1). Then the nitrogen gas was bubbled into the autoclave for three minutes in order to replace the interior air by N_2_, and the initial pressure was kept at 2.0 MPa. After that, the reactor was heated to 140 °C and kept for 1 h. When the reaction was finished, the reactor was cooled down naturally to room temperature. Then the reactor was depressurized, the mixture was poured out and filtrated, washed with reaction solvent and deionized water three times, and was then dried overnight at 100 °C. Finally, the pretreated bamboo was stored under a seal at room temperature after drying, and the dry weight fraction of cellulose in the pretreated biomass was 77.9% [51].

### 3.2. Preparation of Modified Magnetic Fe_3_O_4_ Nanoparticles

Magnetic particles were prepared by the conventional co-precipitation method [35]. In brief, 0.74 g (3.7 mmol) FeCl_2_·4H_2_O and 1.22 g (7.5 mmol) FeCl_3_ were dissolved in 25 mL deionized water under nitrogen with vigorous stirring. Then, 7 mL 30% NaOH was added dropwise, until a black precipitate at room temperature was obtained. The obtained magnetite precipitates were washed several times with deionized water until a pH value of 7 was obtained, and were then dried at 100 °C for 2 h. The magnetic nanoparticles were dissolved in 2.5 mL methanol with 25 μL deionized water and 100 μL APTES, and the mixture was sonicated for 30 min. After that, 1.5 mL glycerol was added, and the solution was refluxed at 90 °C for 6 h with maximum mechanical agitation. Then, the particles were separated from the mixture by the permanent magnet and were washed several times with methanol and deionized water. Finally, the nanoparticles were lyophilized and stored under a seal at room temperature.

### 3.3. Preparation of CLEAs and Magnetic CLEAs of Cellulase

The CLEAs of cellulase were prepared by a conventional method by Šulek et al. [52]. The precipitant (4.5 mL) was added into 0.5 mL of cellulase solution (2 mg/mL, 0.1 M acetate buffer solution, pH 5.0). After keeping the mixture stirring for 30 min at 4 °C, glutaraldehyde was added slowly to the final concentration of 2.0% *v/v* and stirred for 3 h at 30 °C. After that, the suspension was diluted with acetate buffer and centrifuged at 10,000 rpm for 5 min. The precipitate was washed three times by acetate buffer and deionized water, lyophilized, and finally stored at 4 °C.

For the preparation of magnetic CLEAs of cellulase, 5 mg amino functionalized magnetite nanoparticles were mixed with 0.5 mL of free cellulase solution (2 mg/mL, 0.1 M acetate buffer solution, pH 5.0) and were shaken for 30 min under 30 °C. Then, 4.5 mL precipitant was added into the mixture with stirring at 4 °C for 30 min. After precipitation of cellulase, gluaraldehyde was added slowly to the suspension and stirred at 30 °C for 3 h. The suspension was then diluted with acetate buffer and washed three times by acetate buffer and deionized water, lyophilized, and was finally stored at 4 °C.

### 3.4. Protein Binding Ratio and Enzyme Activity Measurements

The protein estimation of the binding efficiency after immobilization was performed according to the Bradford protein assay method [53]. The protein binding ratio was calculated from the following equation:

Protein binding ratio (%) = (amount of protein binded)/(amount of protein added) × 100%,(1)

The cellulase activity was determined by measuring the amount of released glucose during the hydrolysis of sodium carboxylmethyl cellulose (CMC). Cellulase solution (1 mL) and 1 mL of 1% CMC solution (dissolved in 0.1 M acetate buffer, pH 5.0) were incubated at 50 °C for 0.5 h. The glucose produced was measured to calculate the activity by DNS assay [54]. One unit (U) of cellulase activity is defined as the amount of cellulase producing 1 μmol glucose per minute. The specific activity of the free or immobilized cellulase is defined as the amount of glucose produced (in μmol) per milligram of protein used over time. All experiments were repeated at least three times.

### 3.5. Characterization

#### 3.5.1. Fourier Transform Infrared Spectroscopy

The FTIR spectra were recorded on samples in KBr pellets using a Shimadzu FTIR-4200 spectrometer in the frequency range (4000–400 cm^−1^). The samples were mixed with 1% (*w/w*) KBr, and the analysis was performed at 10 scans per second with a resolution of 4 cm^−1^.

#### 3.5.2. Scanning Electron Microscope

The morphology and size of the particles were viewed in a scanning electron microscope (JSM 7500F, JEOL, Tokyo, Japan). The samples were freeze-dried and coated with gold before analysis. The resolution of the SEM was 1.0 nm and the acceleration voltage was 15.0 kV.

#### 3.5.3. UV-Vis

A TU-1901 model UV-Vis double beam spectrophotometer (Beijing Purkinje General Instrument Co., Ltd., Beijing, China) was used to obtain the absorbance of the protein and glucose at 595 nm and 540 nm, respectively.

#### 3.5.4. High Performance Liquid Chromatography

The hydrolysis products of biomass were analyzed by a Waters e2695 HPLC, with an aminex HPX-87 column (Bio-Rad, Hercules, CA, USA) and shodex 101 Refractive Index Detector (RID, Shodex, Tokyo, Japan). H_2_SO_4_ (0.005 M) was used as an eluent at a flow rate of 0.6 mL·min^−1^, the temperature of column oven was 50 °C, and the detector was 35 °C. The products were quantified by the external standard method.

### 3.6. Optimization for Magnetic Cellulase CLEAs Preparation

Acetonitrile, *iso*-propanol, *tert*-butanol, PEG1000 (100% *w*/*v*) and a saturated ammonium sulfate solution were used to precipitate cellulase. The preparation of magnetic cellulase CLEAs was also performed without any precipitant for reference.

To obtain the optimal concentration of the cross-linker, varying final concentrations of glutaraldehyde in the range of 0.2%–4.0% *v/v* were used. Subsequently, varied concentrations of cellulase were added with the constant MNPs during cross-linking, and the ideal weight ratios from 1:10 to 1:1 (*w/w*) of free cellulase and nanoparticles were then determined in order to obtain the maximum activity.

### 3.7. Optimal Conditions for Cellulase Activity

The effect of pH on the activity was evaluated by using buffers of varied pH (3–8). In the pH range of 3–5 a 0.1 M citrate buffer was used, and in the pH range of 6–8 a 0.1 M phosphate buffer was used in the CMC assay. For the optimal temperature of the free cellulase and immobilized cellulase, the activity was determined at different temperatures (30–80 °C) in 0.1 M acetate buffer.

### 3.8. CMC Reusability Study

To analyze the reusability of the immobilized enzyme, the immobilized cellulase was recycled in the CMC hydrolysis reaction 6 times. The immobilized cellulase was separated by a permanent magnet after each cycle, the activity was assayed immediately, and the particles were washed 3 times with acetate buffer (0.1 M, pH 5.0) and deionized water. Then the immobilized cellulase was resuspended again to begin another reaction cycle. The initial activity was defined as 100% and the activity of the recycle runs were expressed as relative activity.

### 3.9. Hydrolysis of Biomass

Different amounts of pretreated bamboo were dispersed in 2 mL of citrate buffer (0.1 M, pH 5.0), and the free cellulase and immobilized cellulase were added which had the same activities (0.24 U), and the hydrolysis reaction was carried out at 50 °C for 48 h with constant stirring. The glucose produced was estimated by HPLC. The hydrolysis percentage of cellulose was calculated as the hydrolysis yield using the following formula:

Hydrolysis Yield (%) = ([glucose])/(1.11 × *f* × [biomass]) × 100%, (2)

where [glucose] is the concentration (g·L^−1^) of glucose obtained after hydrolysis, 1.11 is the weight factor conversion of cellulose to glucose, *f* is the dry weight fraction of cellulose in the biomass (g·g^−1^), and [biomass] is the concentration (g·L^−1^) of the pretreated biomass [55].

### 3.10. Biomass Hydrolysis Reusability

To analyze the reusability in applications, the immobilized cellulase was recycled in the biomass hydrolysis reaction 4 times. The hydrolysis reaction was carried out at 50 °C for 24 h with constant stirring. After the completion of each cycle, the immobilized cellulase was separated by a permanent magnet, and washed 3 times with acetate buffer (0.1 M, pH 5.0) and deionized water. Subsequently, the immobilized cellulase was resuspended in fresh reaction mixture to begin another cycle of the hydrolysis reaction. The initial activity was defined as 100% and the activity of the recycle runs was expressed as relative activity.

## 4. Conclusions

In this paper, novel magnetic cross-linked cellulase aggregates have been developed and applied for biomass bioconversion. Enzyme immobilization using the CLEA technique combined purification and immobilization into a single unit operation, and the use of magnetic particles provided a good reuse potential and ease of operation. The optimal conditions of immobilization were investigated, and the immobilized cellulase showed a better activity at wider temperature and pH values than that of the free cellulase. The immobilized cellulase also had good CMC reusability which could be easily recycled by magnetic separation with 74% of its initial activity retained after six cycles. The magnetic cellulase CLEAs showed a capacity for the hydrolysis of bamboo, which had an immense potential for the efficient conversion of lignocellulosic biomass.
